# The development and validation of a performance infill tracking system to investigate rotational traction mechanisms on artificial turf surfaces

**DOI:** 10.1038/s41598-025-92768-1

**Published:** 2025-03-16

**Authors:** Harry McGowan, Paul Fleming, David James, James Morris, Steph Forrester

**Affiliations:** 1 School of Architecture, Building and Civil Engineering, Loughborough University, Loughborough, UK; 2 Labosport, Unit 3 Aerial Way, Hucknall, UK; 3 Wolfson School of Mechanical, Electrical and Manufacturing Engineering, Loughborough University, Loughborough, UK

**Keywords:** Engineering, Materials science

## Abstract

The scientific principles governing the generation of rotational traction forces on artificial turf remain poorly understood; as such, a photogrammetry technique has been developed to understand the interactions occurring at the boot-surface interface. Videos were recorded through a transparent test foot during rotational traction testing on an artificial turf surface “seeded” with distinguishable performance infill particles. A novel particle tracking software then measured the movement of seeded particles. To determine the uncertainty in the methodology, a gold-standard measurement system determined the distances between 28 fiducial markers. The same marker-to-marker distances were measured using the particle tracking software. For ten static and ten rotating trials, the random bias in the particle tracking software distances was ± 0.89 mm to ± 1.07 mm, respectively. A pilot study on a third-generation artificial turf surface assessed the software’s ability to track infill particles during rotational traction testing. Trials were conducted at two normal loads; particle positions and angular displacements were successfully measured over 40° of rotation and synchronised with torque, angle, and vertical displacement data. A greater number of infill particles were lost during tracking at lower normal loads. This novel methodology represents a useful development in understanding the generation of traction forces, helping to inform future generations of artificial turf and studded footwear.

## Introduction

Traction refers to the resistive force generated as studded footwear interacts with a penetrable sport surface such as artificial turf^1^. Rotational traction forces have been linked closely to the safety and performance of turf surfaces^2–5^. Excessive rotational traction has been linked to ankle and knee injuries^6^; as such, it is a key parameter mechanically tested by sporting governing bodies such as the Fédération Internationale de Football (FIFA)^7^ to certify turf systems in the laboratory and field.

Currently, the interaction mechanisms involved in the development of traction forces on artificial turf are not well described^1^. Previous research has investigated traction using both mechanical and player testing. Both methodologies have predominantly utilised an empirical approach whereby boot and/or surface variables are manipulated and the subsequent effect on traction is measured. While valuable, this approach appears fundamentally limited; despite decades of research into artificial turf surfaces, the rate of traction related injuries has remained consistent^8–12^. Understanding the fundamental mechanisms involved in the generation of traction forces and the most prevalent variables influencing traction generation should provide a more effective method of reducing injury occurrence while maintaining or improving the design and performance of artificial turf surfaces.

Mechanical test devices record the resistive force generated during the interaction between a studded plate or boot outsole and a turf surface^13–18^. Due to the wide range of bespoke test devices, stud configurations, and normal loads used in previous mechanical studies, drawing direct comparisons between results can be challenging. Player studies record the loading and movement patterns produced by athletes during sport specific movements. The complex interaction between an athlete and the surface, and greater variability in movement patterns produced by athletes, makes it challenging to isolate the effect of individual variables on performance^1,19–22^. Player movement studies have shown that athletes experience less than 12 mm of surface contact displacement during sporting movements^20^. In contrast, the peak torque recorded during mechanical testing using the standardised FIFA test device^7^ occurs at approximately 37° (30 mm) of rotation, hypothesised to be related to stud overlap mechanics^1^.

Both mechanical and player studies have one major challenge when providing evidence for traction mechanisms. There is inherent difficulty in visualising the interaction between the surface and a studded test foot or outsole. The mechanisms of traction have therefore been hypothesised by interpreting the torque–angle profiles recorded during rotational traction testing using mechanical test devices^1,23^. Three mechanisms are currently hypothesised once the test foot is in contact with the surface and begins to displace: an initial bulk shear resistance between the outsole and surface; a dynamic frictional force as the test foot begins to “slide” over the surface; and a resistance to horizontal stud displacement provided by the performance infill layer of the surface system^1^.

The aim of this study was to develop a particle tracking methodology capable of recording the movement of performance infill particles during rotational traction testing using a bespoke automated rotational traction tester (ARTT)^24–26^. Particle tracking methodologies have been widely used in fluid mechanics for over 50 years^27^, with more recent developments including three-dimensional (3D) analysis of aquatic environments^28^ and two dimensional (2D) investigations into the movement of debris after an explosion^29^. The basic methodology is the same for all studies, the material of interest is seeded with marked particles, their movement is recorded by one or more cameras before the target variable, usually displacement or velocity, is interpolated onto a spatial grid^27^. The methodology developed in this study is considered a unique attempt at understanding and quantifying the performance infill movement behaviour around a studded test foot interacting with artificial turf. Such understanding has relevance to both footwear and surface industries for the development of safer and better performing products with more controlled traction properties.

## Methodology

### Testing set-up

**Fig. 1:**
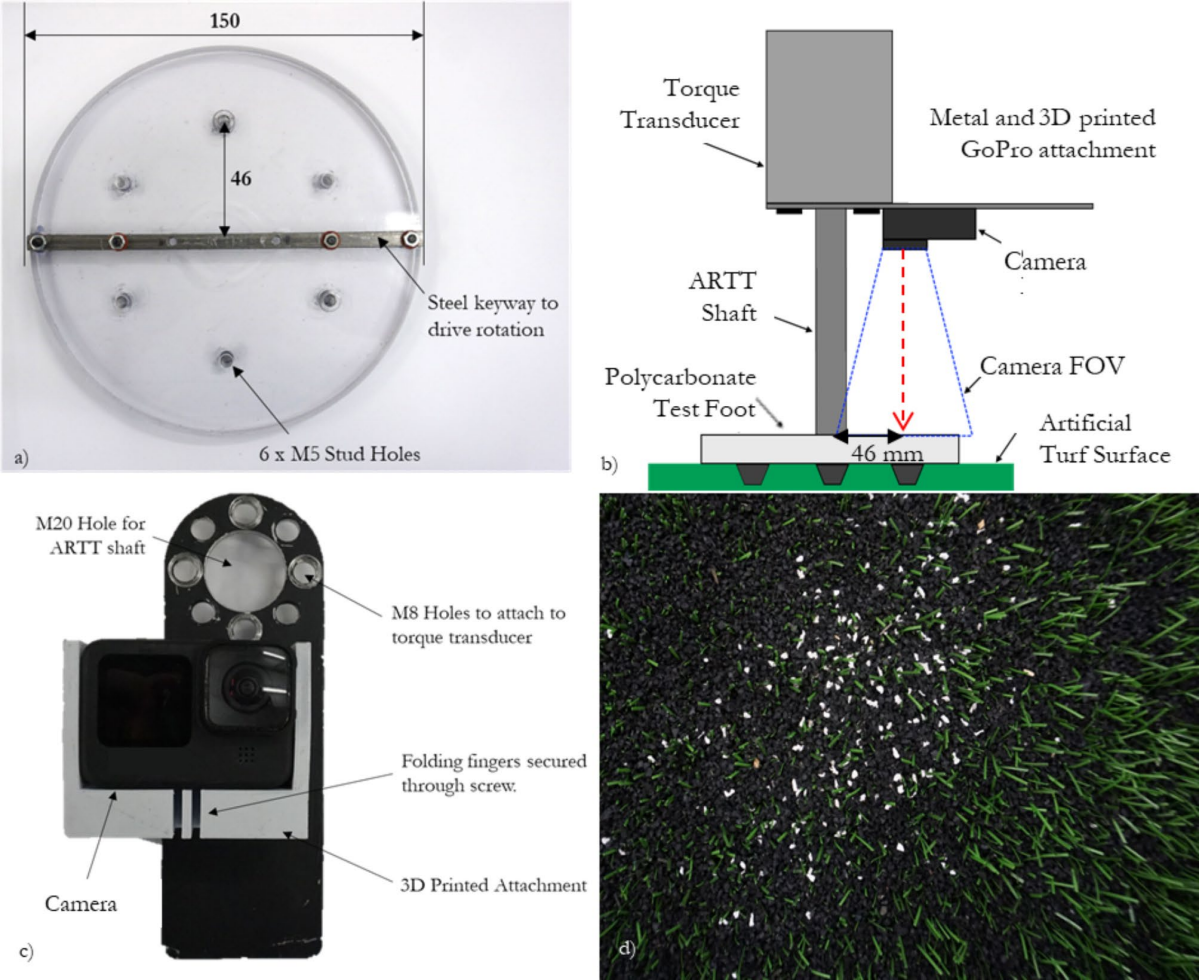
(**a**) The polycarbonate test foot used during rotational traction testing; (**b**) the camera positioning achieved above the polycarbonate test foot; (**c**) the camera, 3D printed holder and metal ARTT attachment used during testing; (**d**) white EPDM particles seeded amongst the black SBR particles and green polyethylene fibres of a typical third-generation artificial turf surface system.

The automated rotational traction tester (ARTT) was adapted to incorporate photogrammetry techniques. A transparent test foot was manufactured from a 10 mm thick sheet of polycarbonate (Fig. 1a). The test foot matched the dimensional specifications in the FIFA guidelines for rotational traction testing^7^. To drive rotation of the test foot, a steel keyway was attached across the centre of the test foot using M3 screws. Six M5 threaded holes were equally spaced on a 46 mm radius from the centre of the test foot to locate the studs, as per the FIFA specifications^7^.

To record videos through the transparent test foot, a single camera set-up was used. For single camera particle tracking, there is a trade-off between the desired resolution and frame rate^22^. To generate comprehensive understanding of infill movement throughout the full range of rotation (120° at 30°/s for the lightweight test device, or at least 45° at 72°/s for the heavyweight test device, as specified by FIFA^7^), it was deemed essential to record particle positions at least every 0.5° of rotation. A previous study identified that peak torque was unaffected by rotational velocities greater than 90°/s^24^. Therefore, the maximum rotational velocity for particle tracking studies was set at 90°/s, giving a minimum camera frequency of 180 frames per second (2 frames per degree of rotation).

The field of view was centred around a single stud and was designed to include half of the test foot (150 mm × 150 mm, 17,700 mm^2^), with three studs spaced 60° apart^7^, and sufficient area outside the test foot for extra markers to synchronise the video and ARTT instrumentation (Fig. 1b).

A low-cost camera (GoPro Hero 10 Black, GoPro, San Mateo, CA, USA) was selected to collect video data as it is capable of recording video footage at 200 frames per second at a resolution of 2704 × 1540 pixels. For the target field of view, this equates to approximately 18 pixels/mm; the performance infill and seeded particles used for tracking were between 1 and 2 mm in diameter, giving sufficient resolution for tracking. Furthermore, the camera is robust, small (71.8 × 50.8 × 33.6 mm) and fits in the desired location below the ARTT’s torque transducer (Fig. 1b).

The camera was attached to the ARTT’s shaft using a bespoke aluminium attachment and 3D printed camera holder (Fig. 1c). The attachment ensured the centre of the camera’s lens was fixed on a 46 mm radius from the centre of the ARTT’s shaft, located directly above one of the six studs on the test foot (Fig. 1b)^7^. Prior to testing, the alignment of the attachment relative to the test foot was assessed using a digital protractor, calibrated on the lab floor which the surface samples were placed.

A styrene butadiene rubber (SBR) infilled surface was prepared specifically to help develop the particle tracking software. The turf surface contained green polyethylene carpet fibres, with black SBR performance infill material. As particle tracking requires a contrast to identify target markers against the background, white ethylene propylene diene monomer (EPDM) particles (Net World Sports, Wales) were seeded on top of the surface system (Fig. 1d). For each trial, more than 70 EPDM particles were seeded on the surface. EPDM was used to seed the surface as white SBR particles in the same size range as the black SBR in the surface system (1–2 mm) could not be sourced. Furthermore, EPDM has similar mechanical properties to SBR^26,30,31^. The EPDM granules were sieved so only particles between 1 and 2 mm were added to the surface^32^.

### Performance infill tracking software

**Fig. 2:**
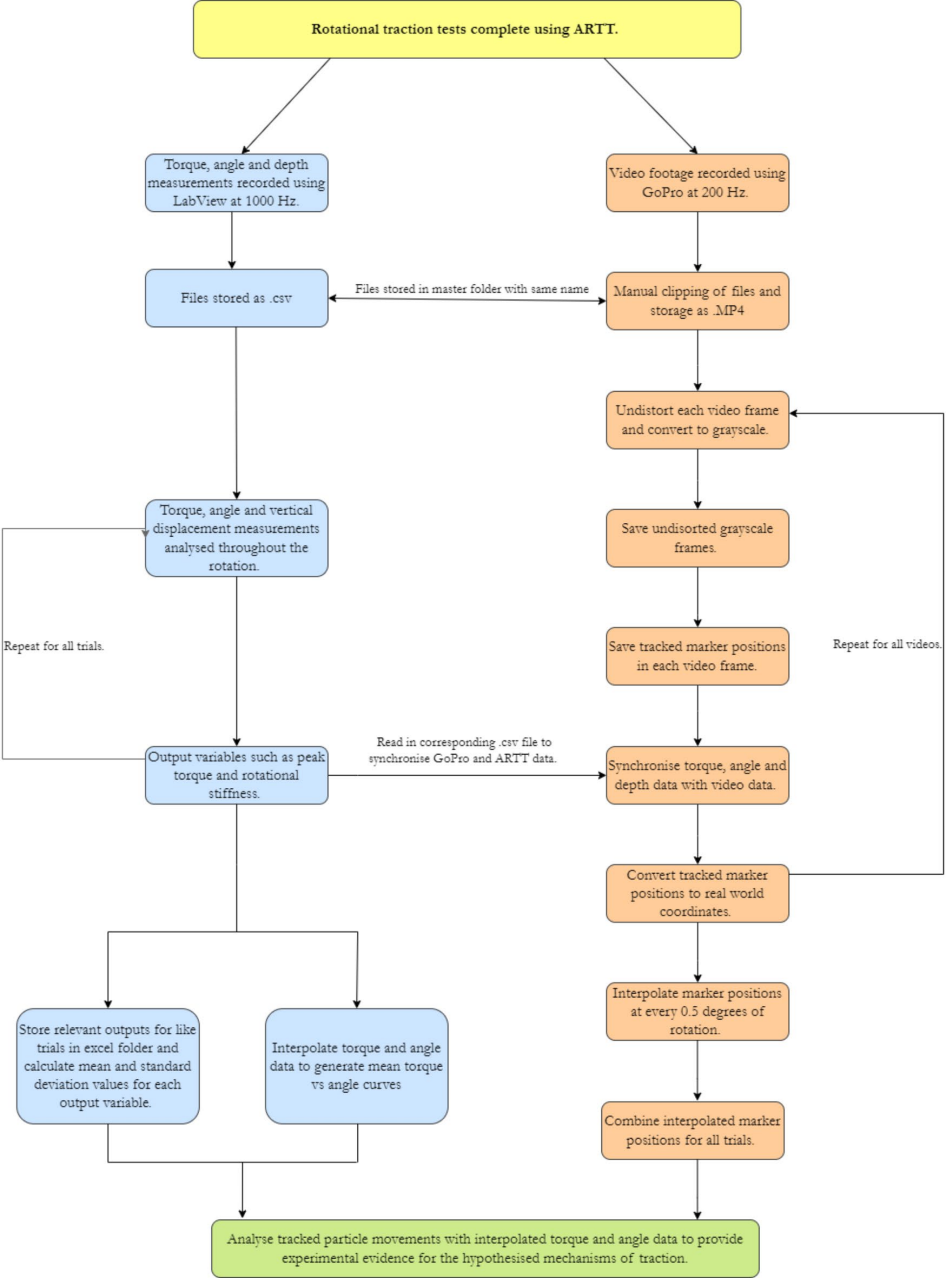
The process for capturing, synchronising, and analysing torque, angle, vertical displacement, and video data. For the validation study the tracking markers were 1–5 mm GOM fiducial markers, for the turf tracking study, markers were seeded EPDM infill particles, a 5 mm white marker placed on the stud centre, and white displacement markers created using a 3D printed part (figure).

A bespoke particle tracking algorithm was developed in MATLAB to analyse each video image and track the movement of white EPDM performance infill particles during rotational traction testing. During a rotational traction test, torque, angle, vertical displacement was recorded using the ARTT’s instrumentation, while video footage was recorded using the camera. The individual trial data was then synchronised to the video footage and analysed during post processing (Fig. 2). Repeat trials were conducted to generate sufficient cumulative particle coverage across the surface. The tracked particle positions from each trial were interpolated and combined; movement of the infill particles was analysed with respect to the mean torque, angle and vertical displacement data recorded during testing.

#### Image processing

Prior to testing, the video camera was calibrated in-situ using the MATLAB camera calibration app^33^. A 15 mm checkerboard calibration panel was placed below a 250 × 500 × 10 mm sheet of polycarbonate. Video footage was recorded through the polycarbonate sheet with the camera in situ. At least twenty frames of video footage were uploaded to the app to calculate the camera’s intrinsic and extrinsic parameters, enabling future videos to be undistorted.

**Fig. 3:**
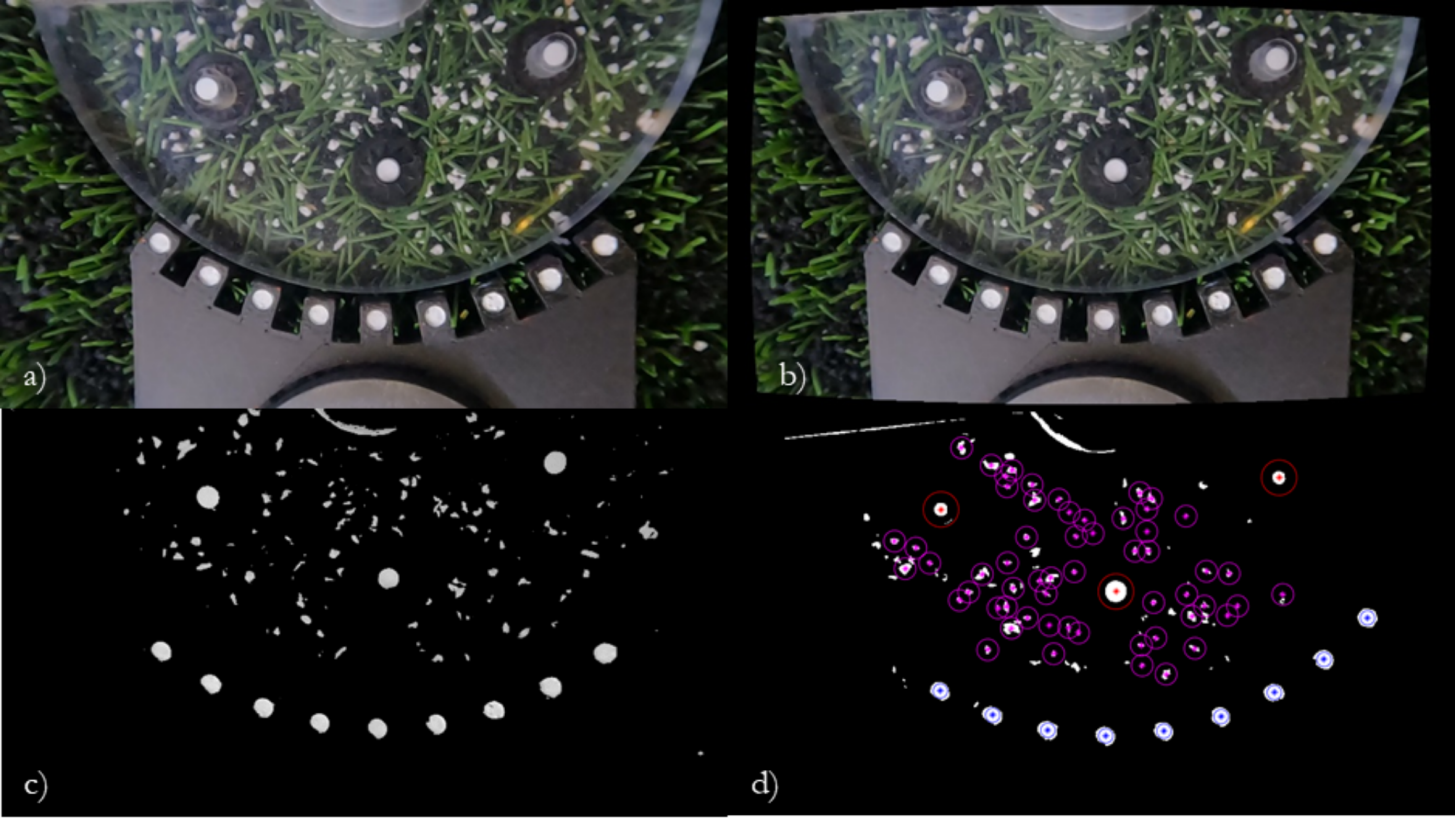
The image processing steps taken to convert the video frames to binary images for tracking. (**a**) a video frame taken from a rotational traction test using the particle tracking set-up. Visible in the field of view are white EPDM particles seeded in an SBR surface, three studs with black backing and white GOM markers to identify their centres, and the data synchronisation tool adjacent to the polycarbonate test foot; (**b**) the undistorted version of the same frame; (**c**) the same frame after the conversion to a binary image; (**d**) the tracked stud markers shown in red, data synchronisation markers shown in blue, and infill particles shown in magenta.

The position of the three studs in the field of view (Fig. 3a) were used to align multiple video trials. Each stud visible in the field of view was tracked during testing; the distance between adjacent studs was calculated in pixels and compared to the known distance between each stud (46 mm), to calculate the scale factor from pixels to millimetres. A data synchronisation tool was 3D printed using polylactic acid (PLA). The data synchronisation tool contained nine white markers (4 mm in diameter) on a black background. Each marker was tracked during data analysis to synchronise the video and ARTT data (Fig. 3a).

The camera parameters calculated during calibration were used to undistort each video frame (Fig. 3b). To improve the contrast between the image background and the seeded particles, each frame was converted to binary using a manually selected threshold (Fig. 3c).

#### Particle selection and tracking

#### First frame

The pixel coordinates of seventy performance infill particles, three stud markers, and nine data synchronisation markers were manually selected in the first frame using a custom graphic user interface (GUI). The MATLAB image processing function *bwconncomp*^34^ was used to find connected components (an area of white pixels denoting a marker) in the first frame. The *regionprops* function^34^ was used to store the area, centroid, and bounding box of each connected component in the first frame. The distance between the manually selected pixel coordinates (*i*)*,* and the connected component centroid in the first frame (*c*) was calculated using Eq. (1). The smallest distance found indicated the particle closest to the user input. Each particle selected was given a unique ID; the corresponding centroid, area and bounding box were stored in a table.

1 $${d}_{ic} = \sqrt{{{(x}_{i} - {x}_{c})}^{2} + {{(y}_{i} - {y}_{c})}^{2} }$$

where ${d}_{ic}$ is the distance between the manually selected particle coordinates and the connected component centroids in the first frame; ${x}_{i}$ and ${y}_{i}$ denote the manually selected coordinates; ${x}_{c}$ and ${y}_{c}$ denote the centroid of each connected component.

#### Subsequent frames

To identify particle locations in subsequent frames, filtration and predictive tracking were used. In the second frame, where no movement was assumed, the particle centroid, area, and bounding box were predicted not to change from the first frame. In subsequent frames, the predicted particle centroid, area, and bounding box were calculated using Eq. (2).

2 $${P}_{n} = {M}_{n-1}+( {M}_{n-1}- {M}_{n-2})$$

where n is the current frame number; P is the predicted particle centroid, area or bounding box, and M is the particle centroid, area or bounding box stored from previous frames. P and M always refer to the same variable.

Predicting the particle coordinates in the following frames lessened the computational burden of the tracking algorithm as a filter removed connected components (particles) located too many pixels away from the predicted position calculation. Only the fifteen closest particles remained; from these fifteen particles, a second filter analysed the connected component centroid, area, and bounding box to those predicted by the tracking algorithm. The best matched connected component was given the same particle ID, and its properties were saved (Fig. 3d). If no particle was found after the second filtration, the particle ID was set to NaN and the particle was deemed “lost”. This process was repeated for each EPDM particle in each frame.

#### Data synchronisation and conversion to real world pixel coordinates

The video footage and ARTT data were aligned by placing the synchronisation tool next to the test foot (Fig. 3). As the test foot and camera begin to rotate, the synchronisation markers remain stationary; in the camera’s field of view however, the synchronisation markers appear to move. The start of the rotation was found by determining the first frame all the synchronisation marker centroids had moved by ≥ 0.5°. The change in angular displacement between frames was calculated using the cosine rule, utilising the synchronisation markers centroid position in two adjacent frames, and the centre location of the shaft.

The first time the angle potentiometer data increased by ≥ 0.5° was identified in the ARTT data and denoted as the start of the trial. These two timestamps were synchronised to signify the start of the trial. The difference in the ARTT and camera sampling frequency (1000 Hz and 200 fps respectively) was used to match the remaining frames with ARTT data.

The synchronised angle data was used to convert from the camera reference frame to the laboratory reference frame for the infill particles, stud, and synchronisation markers. The coordinates of each particle and marker were first translated by the X and Y pixel coordinates of the shaft, aligning the centre of rotation to the image origin. Using the angle of rotation recorded by the ARTT, a rotation matrix corrected for the rotation of the camera between frames before the rotated centroids were retranslated using the shaft coordinates.

#### Interpolation of multiple trials

Multiple trials are needed to generate sufficient coverage of visible seeded particles across the area of interest under the ARTT’s test foot. The particle centroids, torque, angle, and vertical displacement data were estimated every 0.1° for 40° of rotation, using a linear interpolation. This allowed data from multiple trials to be analysed together and incorporated the common angle of peak torque found during rotational traction testing^8^. 

#### Validation of particle tracking software

A comparison between the particle tracking software and an industry standard measurement system quantified the measurement uncertainty of the performance infill tracking software. GOM ATOS (GOM GmbH, 2017) was selected as the industry standard reference measurement technique; the system was calibrated according to manufacturer’s specifications and a measurement uncertainty of 0.018 mm was established^35^.

**Fig. 4:**
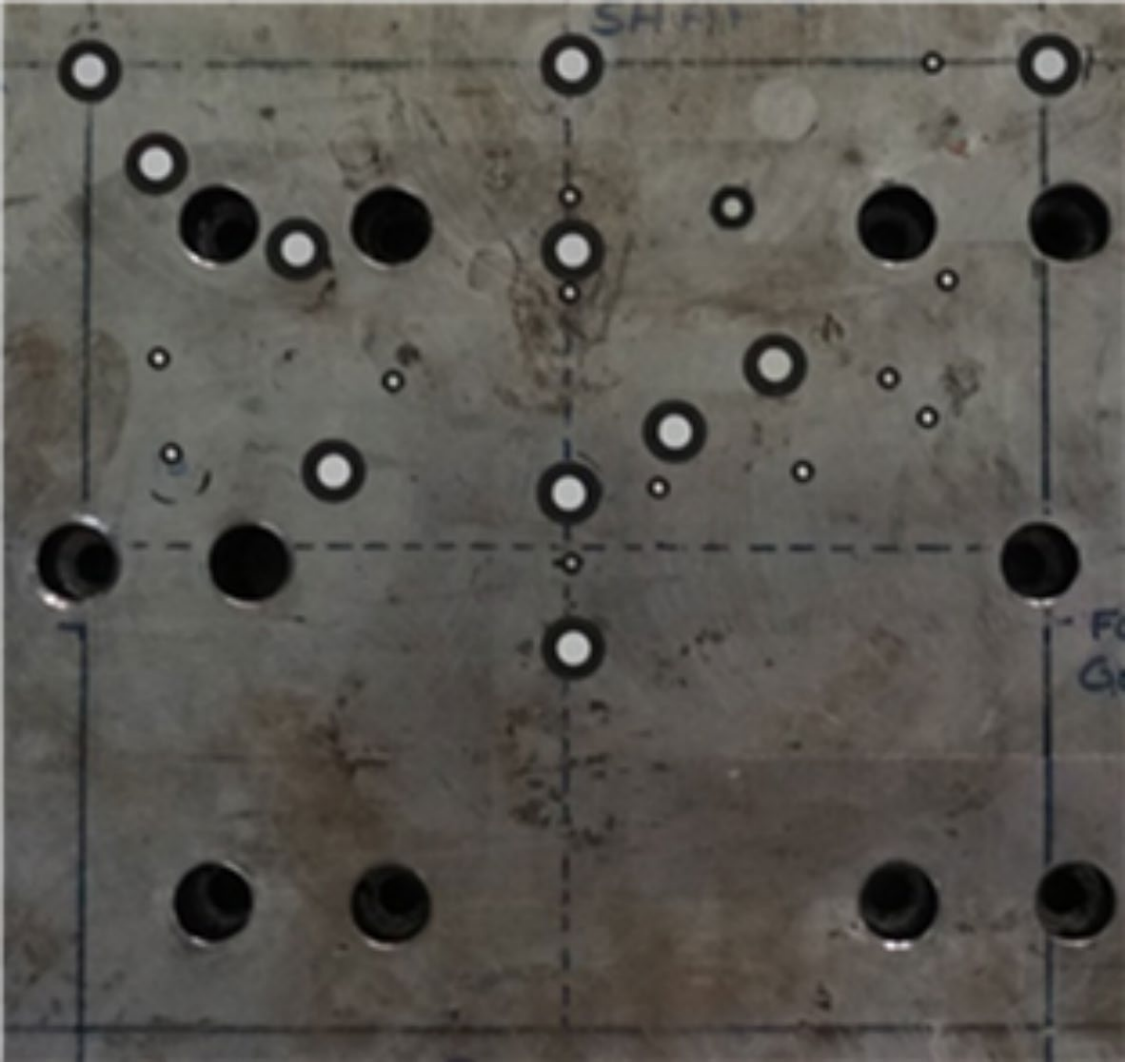
The steel reference object containing 28 fiducial markers, the annotations on the steel block were used by the operator to the size of the FIFA standard test foot.

A reference object was created by placing 28 white-on-black fiducial GOM markers (variably sized markers 1–5 mm in diameter) on a 300 × 300 × 30 mm steel block. The markers were randomly placed within a 150 × 150 mm area before GOM ATOS determined each marker location on the block (Fig. 4). The centre-to-centre distances between all markers (378 inter-marker distances) were calculated using Eq. (3), where ${GSD}_{oq}$ is the calculated distance in millimetres between two markers measured using the GOM ATOS; ${\text{x}}_{o}$ and ${\text{y}}_{o}$ are positions of marker o; ${\text{x}}_{q}$ and ${\text{y}}_{q}$ are the positions of marker q, where markers o and q represent any combination of the 28 markers on the steel block.

3 $${GSD}_{oq} = \sqrt{{({\text{x}}_{o}- {\text{x}}_{q})}^{2} + {({\text{y}}_{o}- {\text{y}}_{q})}^{2}}$$

The polycarbonate test foot was lowered on top of the steel block; ten stationary and rotating videos were then recorded through the test foot. The position of three studs were marked using white markers. Each stud marker and fiducial marker visible below the test foot was tracked using the performance infill tracking software. Only markers that featured below the test foot in the field of view were selected to account for the refractive effect of the polycarbonate test foot. Each stationary video was recorded for one second and each rotating video was recorded over 90° at 30°/s, all at 200 frames per second.

A custom MATLAB script calculated each marker-to-marker distance in pixels. The recorded pixel distance between the stud marker centroids was used to convert from pixels to millimetres. The marker-to-marker distances calculated using the performance infill tracking software were compared to the GOM ATOS reference measurement using Eq. (4). The difference in distance between the particle tracking algorithm and GOM ATOS was recorded for every frame, in each video.

4 $$\Delta {D}_{oq} = \frac{\sqrt{{({\text{x}}_{po}- {\text{x}}_{pq})}^{2} + {({\text{y}}_{po}- {\text{y}}_{pq})}^{2} }}{CF} - {GSD}_{oq}$$

where ${\Delta D}_{oq}$ is the difference between the marker-to-marker distances calculated using the particle tracking algorithm and GOM ATOS reference measure in a given frame and trial; ${\text{x}}_{po}$, ${\text{y}}_{po}$, ${\text{x}}_{pq}$ and ${\text{y}}_{pq}$ are the x and y pixel coordinates of marker o and q respectively, recorded using the performance infill tracking software; $CF$ is the calibration factor calculated by dividing the tracked pixel distance between the studs to the known distance between the studs (46 mm); ${GSD}_{oq}$ is the marker distance in millimetres calculated in the GOM ATOS reference measure. Markers o and q represent any pair of markers visible below the test foot during particle tracking.

The differences between the marker distances calculated using the performance infill tracking system and the reference measure were evaluated using a Bland–Altmann plot. Each plot incorporated the calculated difference in each marker-to-marker distance, for each frame of ten video trials. Stationary trials consisted of 100 frames, whereas rotating trials included 600 frames. The systematic bias, standard deviation ($\sigma$) and 95% upper and lower limits of agreement (ULOA and LLOA respectively) were calculated using Eqs. (5), (6), (7) and (8), respectively.

5 $$Bias =\frac{1}{{m}_{nm}\times {t}_{nt}\times {f}_{nf }} \times \sum_{m f t = 1}^{ {m}_{nm}, { t}_{nt}, { f}_{nf }}{PT}_{mft} - {GOM}_{m}$$

6 $$\sigma =\sqrt{\left|\frac{1}{{(m}_{nm}\times {t}_{nt}\times {f}_{nf })-1} \times \sum_{m f t = 1}^{ {m}_{nm}, { t}_{nt}, { f}_{nf }}{{((PT}_{mft} - {GOM}_{m}) - {M}_{e})}^{2}\right|}$$

7 $$ULOA = (\sigma * 1.96) + Bias$$

8 $$LLOA = \left( {\sigma * 1.96} \right) {-} Bias$$

${PT}_{mft}$ is the marker-to-marker distance calculated using the particle tracking algorithm for a given marker pair, frame, and trial, ${GOM}_{m}$ is the measured marker-to-marker distance calculated from the GOM ATOS reference measure. ${m}_{nm}$ is the total number of marker-to-marker distances; ${t}_{nt}$ is the total number of repeat trials; ${f}_{nf}$ is the total number of frames in each trial.

### Pilot testing on SBR filled surface

Testing was conducted to analyse the software’s ability to track performance infill particles during rotational traction testing. A 1 m^2^ section of artificial turf carpet (50 mm pile height) was infilled first with 8 kg of silica sand (6 mm depth) and 16 kg of SBR (33 mm depth), producing a total infill depth of 39 mm. The surface construction was typical of an artificial turf surface field installation. Construction followed the procedure outlined in the FIFA Handbook of Test Methods; the surface was conditioned prior to testing using a rake and 50 passes of a weighted studded roller^7^.

Eighteen rotational traction tests were conducted at two different normal loads, 177 N and 647 N, generated using static masses. After nine trials had been conducted, the surface was reconditioned (re-raked and rolled 50 times using the studded roller^7^). Normal load has previously been demonstrated to greatly affect the traction forces generated during testing^13–19,26^. The normal load of 177 N was selected as it is the minimum load possible on the ARTT, the load of 647 N was used in a previous study and had produced significantly higher peak torque values than testing conducted at 177 N^26^. Moreover, higher normal loads are more representative of player loading during sporting movements such as cutting and kicking a ball^19–21^.

Prior to the first nine trials, white EPDM particles were scattered randomly on the surface sample below the polycarbonate test foot (Fig. 3). The number of EPDM particles below the test foot after reconditioning was visually assessed, before more white EPDM particles were randomly scattered on top of the surface as required.

The ARTT’s test foot was suspended above the surface at a height of 60 mm using two electromagnets. When the electromagnets were switched off, the test foot dropped onto the surface generating an impact force that ensured full stud penetration into the surface and followed the FIFA standards for rotational traction testing^7^. Rotational traction testing was conducted at 30°/s over 90  of rotation, programmed using the ARTT’s motor control and driven using a timing belt and pulley system^7^. After each drop, the camera’s positioning and alignment were checked.

Video footage was recorded at 200 fps in 2.7 K resolution. Torque, angle, and vertical displacement data was recorded using the ARTT’s instrumentation at 1000 Hz^24–26^. Each video was manually trimmed to the start and end of the rotation (approximately 3 s for a 90° rotation at 30°/s). Seventy EPDM particles, three stud markers, and nine synchronisation markers were manually selected in the first frame (Fig. 3). The start of each trial was identified in the camera footage by finding the first frame all synchronisation markers had moved ≥ 0.5°. This frame was indexed and matched to the first time ≥ 0.5° of displacement was recorded by the ARTT. This process was repeated for every trial conducted, before trials at the same load were combined using a linear interpolation. The angular displacement of each infill marker was calculated from its respective position at the start of the trial using the cosine rule. The number of particles lost during each trial was calculated to assess the robustness of the test set-up.

## Results

### Measurement accuracy and precision

**Fig. 5:**
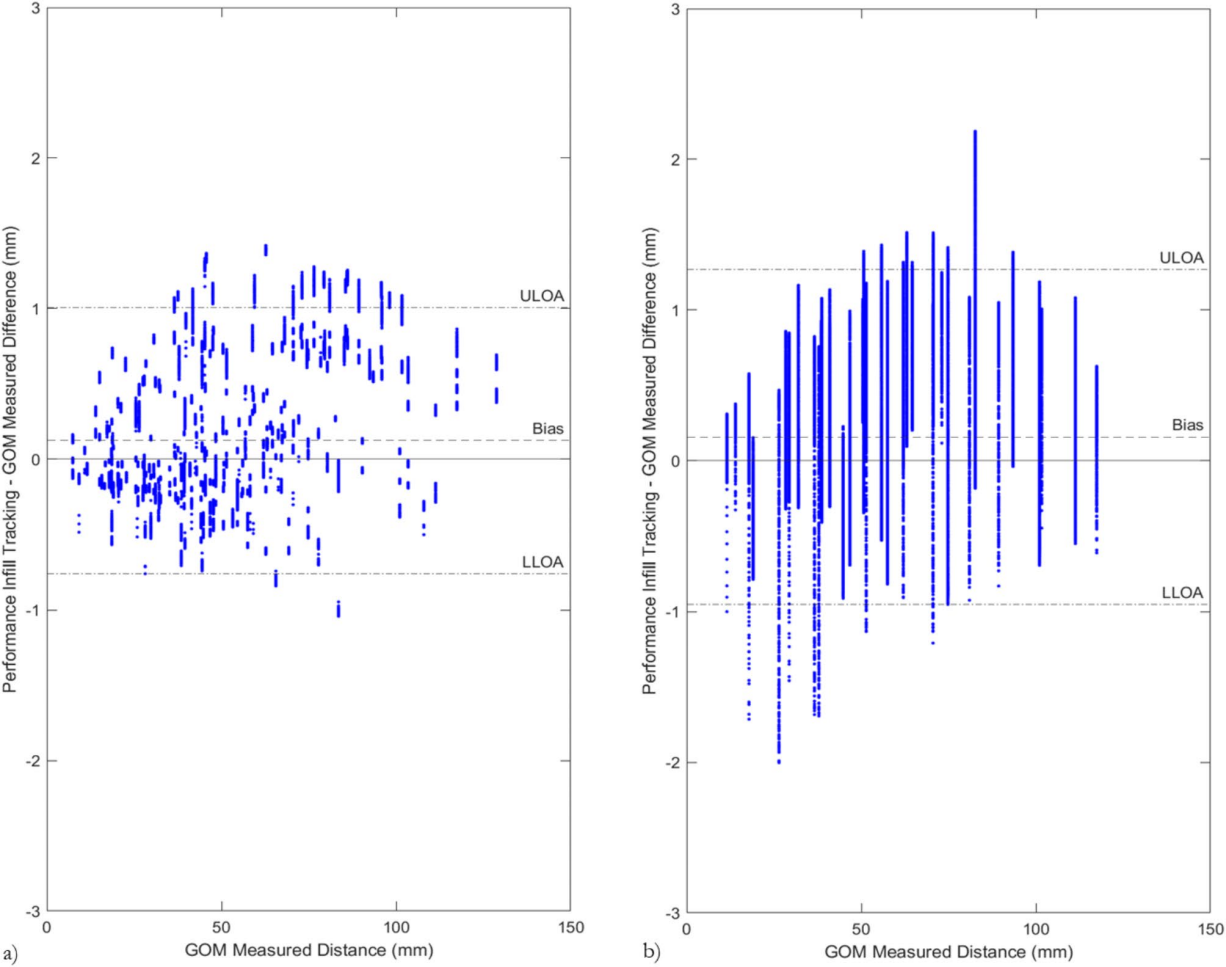
The difference in marker distance calculated between the performance infill tracking system and gold standard (GOM ATOS) reference measurement for (**a**) stationary trials and (**b**) rotating trials. Each blue dot represents the difference between measurement techniques for one marker, calculated from a single frame of one trial.

The difference in measured marker-to-marker distance between the performance infill tracking system and GOM ATOS reference measure is shown in Fig. 5. The systematic and random bias, as well as upper and lower limits of agreement (ULOA and LLOA respectively) are shown in Table 1. In a rotating field of view the uncertainty of the particle tracking system is only slightly degraded. The spread of differences shown in Fig. 5b compared to Fig. 5a indicates that the calculated marker-to-marker distances change more in a rotating field of view, compared to the stationary field of view.

**Table 1:** The systematic bias, random bias, upper, and lower limits of agreement for the ten stationary and rotating video trials analysed during validation testing of the performance infill tracking software.

	Systematic bias (mm)	Random bias (mm)	Upper limit of agreement (mm)	Lower limit of agreement (mm)
Stationary videos	+ 0.12	± 0.89	+ 1.01	− 0.77
Rotating videos	+ 0.16	± 1.07	+ 1.23	− 0.91

### Pilot testing 

**Fig. 6:**
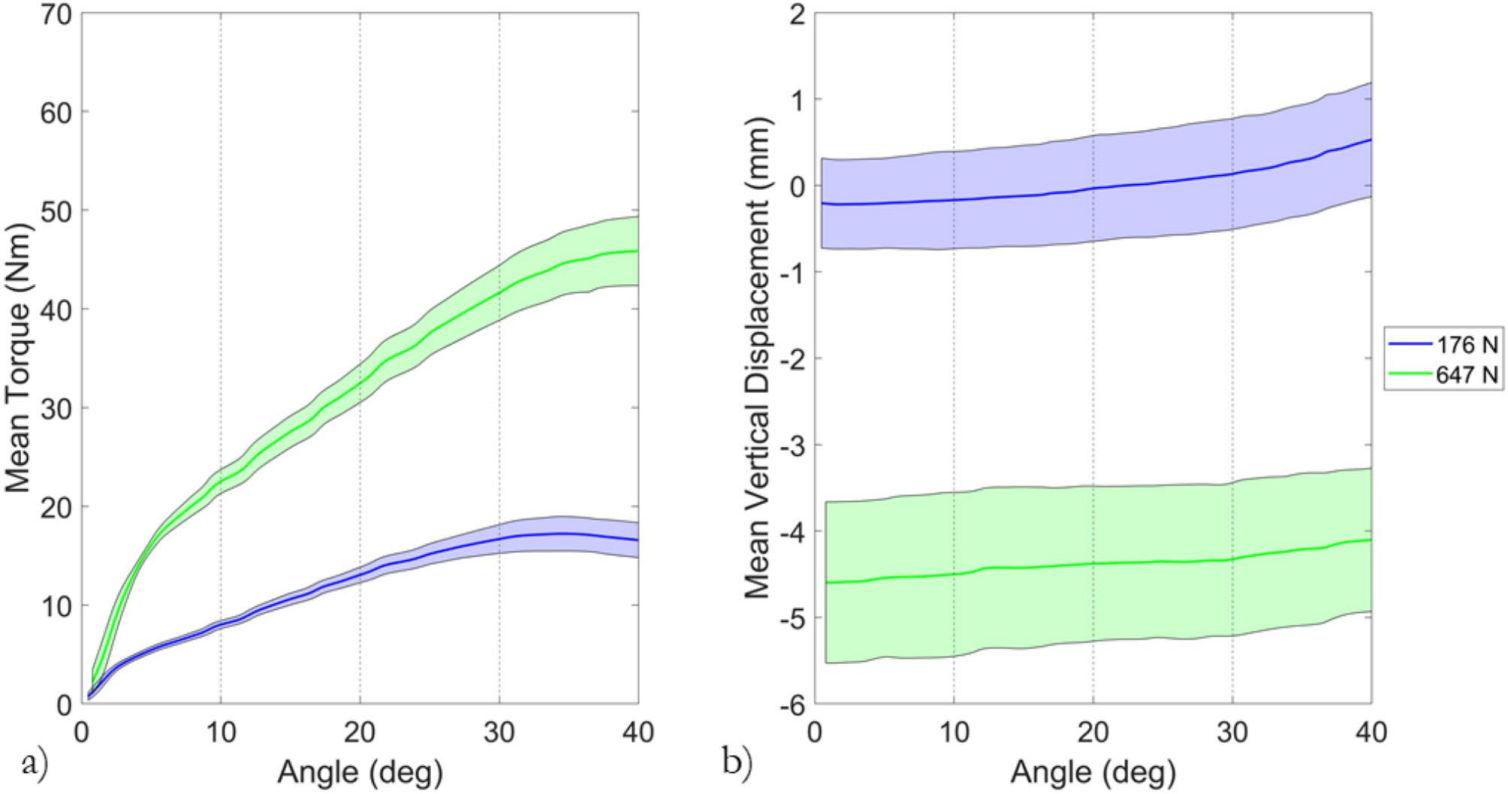
The mean (**a**) torque versus angle and (**b**) vertical displacement for eighteen trials conducted at 177 N (blue) and 647 N (green) of normal load. The shaded area on both graphs indicates the standard deviation calculated from the eighteen trials conducted at each normal load. For the vertical displacement (**b**), a displacement of 0 mm indicates the top of the surface with no load; larger negative magnitudes indicate greater compression of the surface below the test foot.

For eighteen repeat trials at 177 N and 647 N, the mean torque against angle, and vertical displacement against angle plots are shown in Fig. 6. The torques achieved at 647 N were approximately 2.5 times greater than those at 177 N. The mean vertical displacement shows that testing at 647 N produced approximately 4.5 mm more compression of the surface below the test foot than at 177 N.

**Fig. 7:**
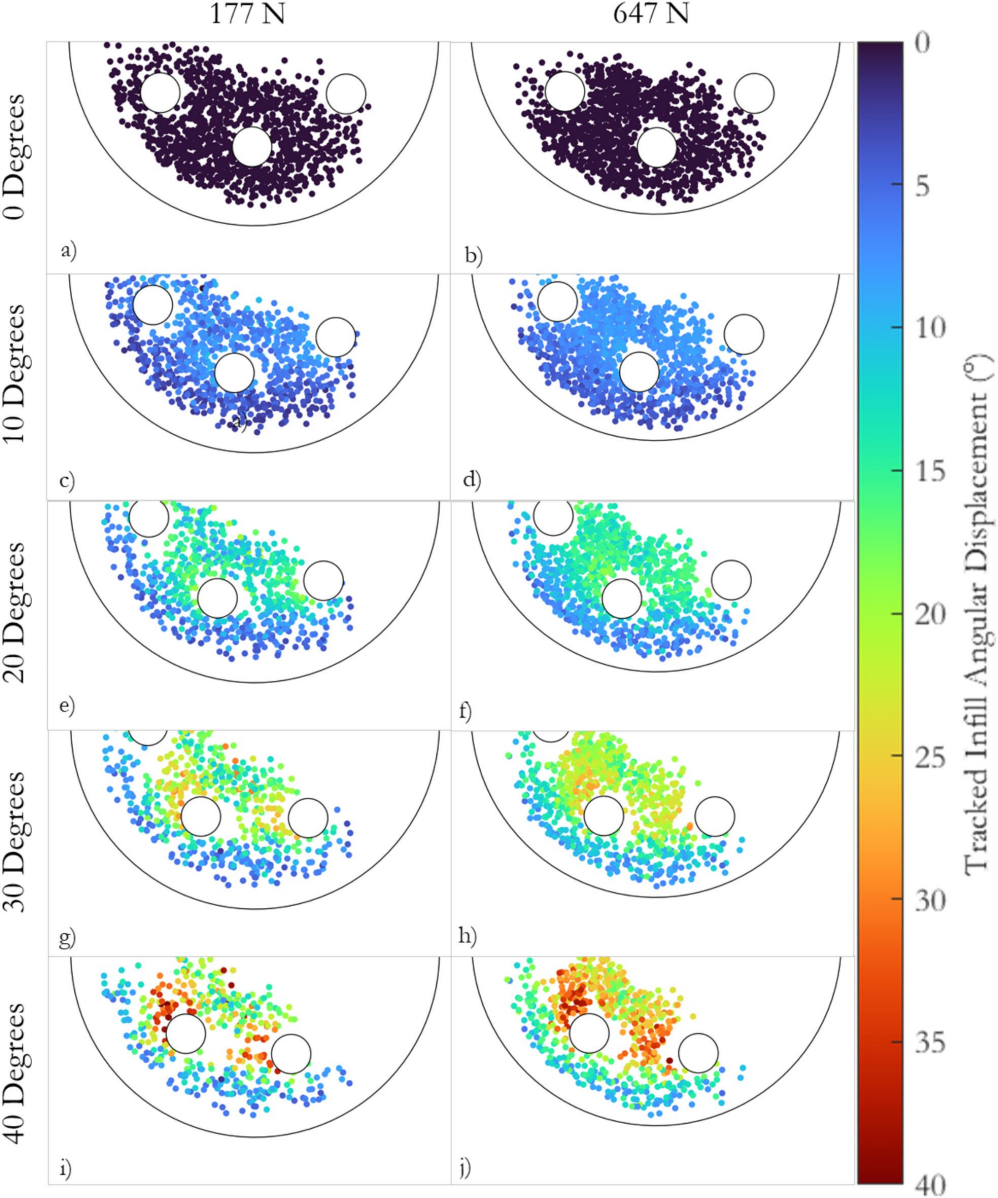
The tracked particle positions for trials conducted at 177 N and 647 N of normal load at (**a**) and (**b**) 0°; (**c**) and (**d**) 10°; (**e** and **f**) 20°; (**g** and **h**) 30°; (**i** and **j**) 40° of rotation, respectively. The colour scale to the side of the figure indicates each particles angular displacement from the start of the trial; each infill marker is coloured with respect to the angular displacement from the start of the trial, calculated using the particle tracking code. Also shown in the figure are the three studs in the field of view, denoted using white circles with a black outline. The edge of the test foot is shown using a black outline.

The tracked performance infill particles positions and colour-coded angular displacements, stud marker positions, and the edge of the test foot are shown in Fig. 7a–j. At 10° of rotation (Figs. 7c and 7d), most particles had displaced between 5° and 10° at both normal loads. At 40° of rotation, greater infill particle displacement was visible on the path of each stud; the infill particles ahead of each stud had displaced approximately 30° to 40°, while particles to the edge of the test foot remained at between 5° and 10° of angular displacement.

**Fig. 8:**
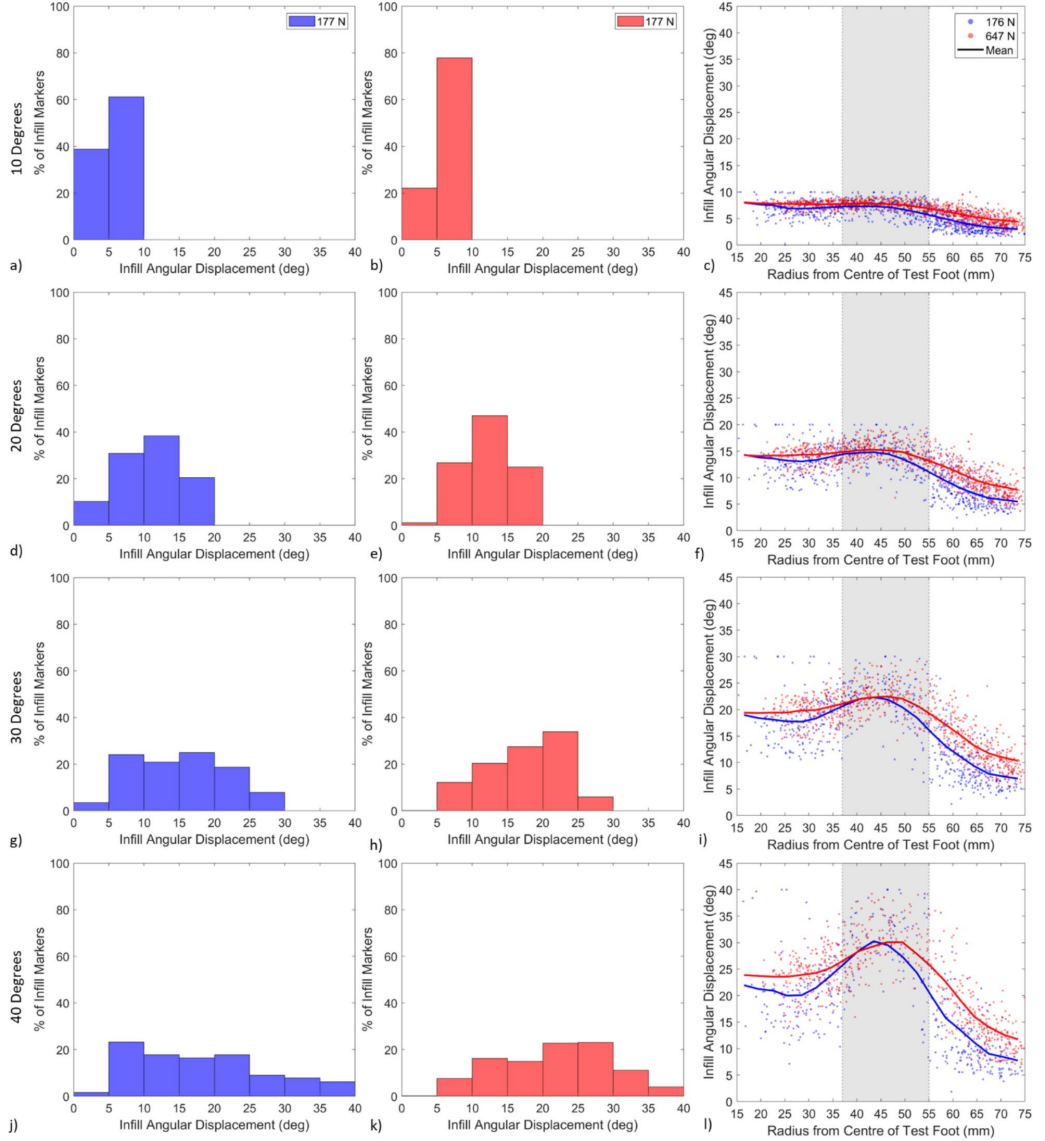
Each histogram shows the distribution in infill material angular displacement at a given test foot angular displacement of 10°, 20°, 30° and 40° of rotation. The scatter plots indicate the relationship between infill angular displacement and each infill markers radial position at 10°, 20°, 30° and 40° of rotation. Grey shading is used in the scatter plots to indicate the width of the stud. The blue (177 N) and red (647 N) lines indicate the calculated mean infill angular displacement at each normal load; a five-point moving average was used to smooth the curve.

To quantitatively analyse the movement of tracked infill particles, histograms and scatter plots were created at 10°, 20°, 30° and 40° of rotation (Fig. 8). The histograms present the distribution of angular displacements in the tracked infill particles at each stage of rotation. The scatter plots show the relationship between the angular displacement of each infill particle and its radial position from the centre of the test foot. The mean infill particle displacement was calculated across the radius of the test foot using a moving average, presented for both normal loads in each scatter plot.

The histograms and scatter plots reinforce the observations presented in Fig. 7; at 10° of rotation (Fig. 8a,b) all the infill particles displace similarly to the studs, while a broader range of infill particle displacements were found as the angle of rotation increased (Figs. 8d,e,g–k).

The scatter plot at 10° of rotation (Fig. 8c) shows a consistent relationship between infill particle angular displacement and infill radial position across the whole test foot. The mean infill angular displacement curve of both normal loads was relatively flat across the radius of the test foot, suggesting the displacement of infill particles was the same across the test foot radius, with a slight decrease at large radii.

As the angle of rotation increased (Fig. 8f,i and l), an increasing peak in infill angular displacement was observed in the radial zone occupied by the studs, highlighted in grey. The infill angular displacement at small radii (centre of the test foot) was closer to the observed peak compared to the displacements measured at larger radii (edge of the test foot).

The scatter plots suggest the infill particles on the path of each stud displace similar magnitudes at both normal loads, supplementing the findings in Fig. 7. Comparable peaks in the radial zone occupied by each stud were found at every angle of rotation, for both normal loads (Fig. 8c,f,i and l). The drop off in infill angular displacement at the larger radii (edge of the test foot) was more pronounced at 177 N compared to 647 N.

The histograms at each angle of rotation confirm the increased infill angular displacement at higher loads. A greater percentage of the infill particle distribution exhibited larger infill angular displacement at 647 N of normal load compared to 177 N (Fig. 8a,b,d,e,g–k). For example, at 10° of rotation (Fig. 8a,b), approximately 78% of the infill material displaced between 5° and 10° for testing at 647 N, whereas at 177 N, this percentage was lower at approximately 61%. At 40° of rotation, 60% of the infill had an angular displacement greater than 20° at 647 N, whereas at 177 N only 41% of the infill had displaced 20° or more.

The number of particles initially selected in the GUI, and number of particles tracked and lost at 0°, 10°, 20°, 30° and 40° of rotation are shown in Table 2. At both normal loads, particles were successfully tracked throughout the rotation. Some of the 1260 particles selected in the GUI input were lost due to sub-optimal thresholding when creating binary images and sub-optimal lighting in the laboratory.

**Table 2:** The number of particles remaining, lost, and equivalent percentage of remaining particles from those manually selected in the GUI input at 0°, 10°, 20°, 30° and 40° of rotation, for the eighteen trials at 177 N and 647 N of normal load.

Rotation angle (°)	177 N			647 N		
	No. particles remaining	No. particles lost	% of particles remaining	No. particles remaining	No. particles lost	% of particles remaining from 0°
GUI Input	1260	0	100	1260	0	100
0	1091	169	86%	1166	94	93%
10	888	372	70%	1052	208	83%
20	683	577	54%	913	347	72%
30	545	715	43%	734	466	58%
40	424	836	34%	630	630	50%

During tracking, particles were lost due to being obscured from the field of view, colliding with other EPDM particles, or simply rotating out of the cameras field of view. Both prior to and during the rotation, a greater number of particles were lost at 177 N compared to 647 N.

## Discussion

This study aimed to develop and validate a test methodology to investigate the movement of infill particles during rotational traction testing on an artificial turf surface. A camera recorded video footage through a transparent, polycarbonate test foot at 200 frames per second and a resolution of 2704 × 1540 pixels (18 pixels/mm). The area visible in the camera’s field of view encompassed half of the ARTT’s test foot, with one stud positioned centrally, and two further studs visible. To maintain the field of view around the central stud, the camera was securely attached to, and rotated with, the ARTT’s shaft during testing. A rotation matrix converted the camera reference frame to the laboratory reference frame. Synchronisation markers were used to align the start of each trial in the video footage with the ARTT’s instrumentation. The measurement uncertainty in the test set-up was investigated and a pilot study conducted. The movement of performance infill material was recorded during eighteen rotational traction tests at two normal loads. The test methodology provided experimental evidence for the interaction behaviours between the performance infill layer, studs and test foot for the first time in literature.

### Measurement uncertainty

The uncertainty of the measurement methodology was investigated by comparing the measured distance between pairs of markers using the performance infill tracking system to a gold standard reference measurement (GOM ATOS). The calculated systematic bias and limits of agreement were slightly increased for rotating video trials (Bias =  + 0.16 mm; ULOA =  + 1.23 mm; LLOA = − 0.91 mm) compared to stationary trials (Bias =  + 0.12 mm; ULOA =  + 0.89 mm; LLOA = − 1.01 mm).

Whilst comparable particle tracking systems are hard to find due to the unique objectives of the test methodology, previous studies have used a similar calibration method to record high speed observations of the boot-surface interaction^36^. During a rotation of 40° (which typically incorporates the achievement of peak torque during FIFA specified rotational traction testing^1,7^), the studs displace approximately 32 mm. Thus, a random bias of ± 1.07 mm is considered suitably small to measure the distribution of particle displacements during rotational traction testing. The slightly lower accuracy during rotational movements may be due to the increased refractive effect of the polycarbonate test foot as markers move through the field of view, and a change in 2D orientation of the tracked markers.

### Pilot study on an artificial turf surface system

During the pilot study, white EPDM seed particles, stud markers, and synchronisation markers were successfully tracked during rotational traction testing at two normal loads, 176 N and 647 N (Figs. 3 and 7). The recorded particle positions were synchronised with the rotation angle, resistive traction torque and change in vertical displacement recorded by the ARTT (Fig. 6). Eighteen repeat trials were conducted and combined to generate sufficient particle coverage and compensate for the loss of particles during tracking (Table 2). The total viewing area of interest below the test foot was approximately 5022 mm^2^. When divided by the 1260 particles selected in the GUI input, the average minimum particle coverage was approximately one particle per 4 mm^2^.

#### Implications for the hypothesised traction mechanisms

In agreement with previous literature, testing at higher normal loads (647 N) produced approximately 2.5 times greater torque values (Fig. 6a)^13–19,26^. A greater vertical displacement, i.e., higher compression of the surface, was observed at the higher normal load as expected for a deformable rubber performance infill material (Fig. 6b)^32^. The video footage and tracked particle movements enabled observation of the interaction between the performance infill material, studs and test foot. Previously, three mechanisms of traction have been suggested: an initial bulk shear resistance provided by the infill material; a dynamic friction resistance as the test foot begins to slide over the surface; and the resistance provided by the performance infill layer to the displacement of the studs^1^.

The initial bulk shear, and resistance to stud displacement mechanisms are critically appraised considering the unique data obtained in this study. At 10° of rotation for both normal loads, most particles experience angular displacements of between 5° and 10° (Fig. 7 and 8). Particles towards the outer edge of the test foot experienced slightly lower displacements. The similarity in infill angular displacement across the test foot provides evidence that the high rate of torque increase observed in the early stages of rotations at both normal loads (Fig. 6a) is related to a bulk shear resistance provided by the performance infill layer (Fig. 8)^1^.

At larger angles of rotation, the infill markers located on the path of each stud show greater angular displacement compared to the markers at either side of the stud (Figs. 7 and 8). At both normal loads, the maximum infill displacement ahead of each stud is of similar magnitude to the angle of rotation during testing (Fig. 7). The infill material towards the centre and edge of the test foot undergoes lower angular displacement than the material on the path of each stud. Linear increases in torque were observed between 10° and the peak torque at both normal loads (Fig. 6a). The increase appears driven by a zone of infill material on the path of each stud generating resistance to stud displacement, agreeing with the previously hypothesised mechanisms of traction by Forrester and Fleming^1^.

The similarity in the infill angular displacements ahead of each stud for both normal loads suggests the mechanisms generating traction forces are consistent and independent of the normal load used during testing (Figs. 7 and 8). The increased torque values generated at 647 N of normal load (Fig. 6a) can therefore be attributed to the increased shear resistance and interparticle forces in the infill material. Increased interparticle forces are generated by the increased compression of the surface under the test foot at higher normal loads (Fig. 6b). During the initial stages of rotation, the increased interparticle forces generate a greater bulk shear resistance, while at larger displacements, the increased interparticle forces provide more resistance to stud displacement in the zone of infill being displaced on the path of each stud (Fig. 6a). Greater overall infill movement was observed at higher normal loads, predominantly towards the centre and outer edge of the test foot, as shown by the histograms and scatter plots in Fig. 8.

#### Implications for the test methodology

Particles were lost during rotation of the test foot at both normal loads examined (Table 2). Over the first 10° of rotation, 30% of particles had been lost at 177 N, while only 17% had been lost at 647 N. At 40° of rotation the number of particles lost increased to 66% and 50% respectively. There are multiple reasons for the loss of tracked, seeded particles with increased rotation angle. Tracked particles rotated out of the field of view; collided with other white EPDM particles thereby being removed during filtration; or were obscured from view by SBR performance infill and carpet fibres.

The pilot study showed increased particle retention was observed at 647 N and at smaller angles of rotation (Table 2). Between 0° and 10° of rotation at 647 N, 94% of the markers initially selected in the GUI input remained. This suggests the test methodology is well suited for investigating loads and displacements more representative of realistic sporting movements. Player ground reaction forces are commonly 1–1.5 bodyweights, plantar pressures typically range from 510 to 873 kPa, and rotation angles of less than 12° have been recorded during stop and turn movements in previous studies^20,37^.

While more particles remained visible during tracking at the higher normal load, it is essential to understand the effect of normal load on traction mechanisms across a range of normal loads. Currently, FIFA standardised testing only includes one normal load of 451 N which fails to capture the full relationship between normal load and rotational traction^7,26^. Although more particles were lost at lower loads, the number of repeat trials can be increased to generate sufficient particle coverage below the test foot.

The tracking system is a 2D representation of the 3D performance infill layer, making drawing conclusions about what is occurring below the boot-surface boundary challenging. The increased infill displacement on the path of each stud is hypothesised to occur throughout the stud depth. Stud length has been shown to increase rotational traction forces, supporting the hypothesis that longer studs displace an increased volume of infill material^13,38^. Previous research has shown two studs positioned orthogonally to the direction of movement increases traction compared to studs on a linear path^8^. It is not currently known how the infill material behaves and interacts around and between each stud. Such stud configurations more closely resemble the outsoles of commercial football boots, as opposed to testing related to the FIFA standards^7^. Insights from these studies can be used to optimise future boot designs and outsole configurations to improve the safety and performance of studded footwear.

Variables related to the surface system, such as different types of performance infill material, surface wear, infill depth and fibre density can also be investigated using the particle tracking methodology^1^. Due to the European ban on the sale of SBR as a performance infill material^39^ it is essential to understand any potential differences in the traction mechanisms between polymeric and organic performance infill materials. Finite element analysis (FEA) models for artificial turf surfaces are also becoming more common in literature^40^. Generating knowledge of the interactions occurring at the boot-surface interface can help validate the accuracy of such models. Understanding and predicting the traction response when multiple variables are manipulated concurrently can ensure future artificial turf surfaces maintain or improve on the current safety and performance standards.

## Conclusion

This novel test methodology is the first in literature developed to record the movement of performance infill particles during rotational traction testing on an artificial turf surface. The systematic and random bias in measurements were + 0.16 mm and + 1.07 mm, respectively. A pilot study was successfully conducted at two normal loads. The movement of 70 EPDM particles was tracked in each of eighteen repeat trials and synchronised with torque, angle and vertical displacement data. The preliminary tracking results support the previously hypothesised mechanisms of traction in terms of an early bulk shear resistance and increased infill displacement in front of each stud at greater rotations. Furthermore, the observed mechanisms remained consistent at both loads tested, suggesting the increased torque recorded at higher normal load is due to increased interparticle forces in the performance infill layer. Insights such as these have the potential to support innovation in artificial turf and studded footwear design to achieve a specific traction response.

## Data Availability

The authors confirm that the data supporting the findings of this study are available within the article and on request from the corresponding author.
